# Detection of Autophagy-Related Gene Expression by Conjunctival Impression Cytology in Age-Related Macular Degeneration

**DOI:** 10.3390/diagnostics11020296

**Published:** 2021-02-12

**Authors:** Chih-Wen Shu, Youn-Shen Bee, Jiunn-Liang Chen, Chui-Lien Tsen, Wei-Lun Tsai, Shwu-Jiuan Sheu

**Affiliations:** 1 Institute of Biopharmaceutical Sciences, National Sun Yat-Sen University, Kaohsiung 80424, Taiwan; 2 Institute of Biomedical Sciences, National Sun Yat-Sen University, Kaohsiung 80424, Taiwan; 3 Department of Biomedical Science and Environmental Biology, Kaohsiung Medical University, Kaohsiung 80708, Taiwan; 4 Department of Ophthalmology, Kaohsiung Veterans General Hospital, Kaohsiung 81362, Taiwan; ysbee@vghks.gov.tw (Y.-S.B.); jlchen@vghks.gov.tw (J.-L.C.); cltsen@vghks.gov.tw (C.-L.T.); 5 Department of Internal Medicine, Kaohsiung Veterans General Hospital, Kaohsiung 81362, Taiwan; tsaiwl@yahoo.com.tw; 6 School of Medicine, National Yang-Ming University, Taipei 11221, Taiwan; 7 Department of Ophthalmology, Kaohsiung Medical University Hospital, Kaohsiung 80708, Taiwan; 8 School of Medicine, Kaohsiung Medical University, Kaohsiung 80708, Taiwan

**Keywords:** age-related macular degeneration, autophagy, GABARAPL1, impression cytology, oxidative damage

## Abstract

Purpose: To investigate the association of autophagy-related gene expression with age-related macular degeneration (AMD). Methods: Patients with AMD were recruited for analysis by conjunctival impression cytology. mRNA was assessed by real-time polymerase chain reaction (RT-PCR) to evaluate whether the expression of 26 autophagy-related genes (ATGs) was correlated with AMD. Further studies on cell viability and autophagic flux in response to oxidative stress by H2O2 were performed in human retinal pigment epithelial (RPE) cell lines based on the results of impression cytology. Results: Both the neovascular AMD (nAMD) and polypoidal choroidal vasculopathy (PCV) groups had significantly higher mRNA levels of gamma-aminobutyric acid receptor-associated protein-like 1 (GABARAPL1) and microtubule-associated proteins 1A/1B light chain 3B (MAP1LC3B) than the control group, but there was no significant difference between these two groups. Age difference existed only in the AMD group. GABARAPL1 and MAP1LC3B mRNA expression increased significantly after acute oxidative stress in adult retinal pigment epithelial (ARPE-19) cells. Cell viability significantly increased and decreased in the cells harboring GABARAPL1 expression vector and silenced with siRNA against GABARAPL1, respectively, during short-term oxidative stress, whereas viability increased in the GABARAPL1-silenced cells after long-term oxidative stress. Silencing GABARAPL1 itself caused a reduction in autophagic flux under both short and long-term oxidative stress. Conclusion: Our study showed the possibility of assessing autophagy-related gene expression by conjunctival impression cytology. GABARAPL1 was significantly higher in AMD. Although an in vitro study showed an initial protective effect of autophagy, a cell viability study revealed the possibility of a harmful effect after long-term oxidative injury. The underlying mechanism or critical factors require further investigation.

## 1. Introduction

Advanced age-related macular degeneration (AMD) is a common cause of uncorrectable severe vision loss in elderly people worldwide [1,2,3]. Polypoidal choroidal vasculopathy (PCV), more prevalent in the Asian population, has been considered a variant form of neovascular AMD (nAMD). Many characteristics specific to PCV have indicated that PCV might be a different disease entity from typical nAMD, including the clinical characteristics such as orange color nodule-like lesion with massive hemorrhage, and a specific branch vascular network with polyps in an angiogram. Recent studies in the genetics, imaging and clinical response to treatment suggested differences in pathophysiology between PCV and typical nAMD [4]. While the pathophysiology remains poorly understood, a growing body of evidence indicates that cumulative oxidative stress is related to the underlying mechanism of AMD [5]. Degeneration of retinal pigment epithelial (RPE) cells has been implicated in the early pathogenesis of AMD, although the vision changes result from photoreceptor death in the central retina [6,7]. The cells are responsible for phagocytosis and the degradation of shed photoreceptor outer segments. It has been demonstrated that these cells die through apoptosis in the eyes of AMD patients [8,9]. 

Autophagy is a self-eating process that degrades dysfunctional proteins and organelles in lysosomes and generates new substrates to be recycled in cells during starvation and stress [10,11]. It plays a beneficial role in the normal homeostasis of the ocular cells, which is involved in the maintenance of outer segment turnover in photoreceptors and protects RPE cells from stress. However, a study showed that autophagic cell death was promoted in retinal ganglion cells during chronic intraocular pressure elevation. The role of autophagy might vary depending on the types of ocular cell or stress. In recent years, the relationship between autophagy and AMD has been reported but is controversial. Autophagy plays a complex role in cells. This pathway can improve cell survival through the recycling of proteins; inhibited autophagy can induce cell death through the activation of apoptosis in RPE cells. However, excessive autophagy might promote cell death in cells under certain conditions, known as type II programmed cell death [12,13,14]. Our previous studies showed that silencing erb-b2 receptor tyrosine-protein kinase 2 (ERBB2) inhibited reactive oxygen species (ROS) production in ARPE-19 cells during oxidative stress. The knockdown of ERBB2 might reduce autophagic cell death in ARPE-19 cells during oxidative stress, suggesting that ERBB2 may be the link between kinases and autophagy in RPE cells, at least in the context of oxidative damage [13]. The role of autophagy in AMD needs further investigation.

Impression cytology is a useful clinical test and is less invasive than biopsy. The technique was used for studying ocular cells [15]. With the improvement of the real-time reverse transcriptase polymerase chain reaction (RT-PCR) method, mRNA expression levels can be evaluated on the ocular surface by using impression cytology specimens [16]. In this study, we first isolated mRNA from patients for conjunctival impression cytology to investigate the autophagy-related gene expression of patients with age-related macular degeneration and further studied these genes in human retinal pigment epithelial (RPE) cells based on the results of impression cytology.

## 2. Materials and Methods

### 2.1. Cell Culture of ARPE Cells

Adult human RPE cells (ARPE-19) were obtained from the American Type Culture Collection (CRL-2302; ATCC, Manassas, VA, USA). Briefly, ARPE-19 cells were cultured in a DMEM/F12 medium (Dulbecco’s modified Eagle’s medium and Ham’s F12 medium) with sodium bicarbonate (1.2 g/L), L-glutamine (2.5 mM), 4-(2-hydroxyethyl)-1-piperazineethanesulfonic acid (HEPES, 15 mM), sodium pyruvate (0.5 mM) and 10% fetal bovine serum (Life Technologies, California, USA) and grown in a humidified incubator at 37 °C with 5% CO2.

### 2.2. Real-Time PCR

We recruited 20 patients with exudative AMD, 22 patients with polypoidal choroidal vasculopathy (PCV) and 27 with cataracts without any fundus abnormalities as controls from the outpatient clinic of the Department of Ophthalmology, Kaohsiung Veterans General Hospital (approved by Institutional Review Board in Jan. 15, 2018; No: VGHKS17-CT12-18). Care was taken to exclude patients who reported any infective or autoimmune diseases, which might interfere with the analysis. Those without sufficient samples for analysis were also excluded. The fundus image analysis and fundus fluorescent angiography were performed by using a digital fundus camera (Visupac 450, Zeiss FF450, Carl Zeiss Meditec AG, Jena, Germany). The grading of AMD used a simplified severity scale for AMD. Written informed consent was obtained from all participants by a consent form approved by the Institutional Review Board for Human Research of Kaohsiung Veterans General Hospital. The procedures used conformed to the tenets of the Declaration of Helsinki.

After topical anesthesia, four pieces of nitrocellulose blotting membrane (Amersham Protran 0.2 µm NC; GE Healthcare Life Sciences, Marlborough, MA, USA) were placed in each quadrant of the bulbar conjunctiva for 15 s. RNA isolation for impression cytology was performed according to the manual instructions of the RNeasy Micro kit (Qiagen, 74001, Germantown, USA ). Briefly, 350 µl of lysis buffer and an equal amount of 70% ethanol were added to the sample to precipitate the nucleic acid. The crude nucleic acid was washed to remove protein and salt. The DNA of the nucleic acid was further degraded with DNase to obtain pure RNA. Total RNA (1 µg), reverse transcriptase (Invitrogen, 18064-014) and a ToolsQuant II Fast RT kit (Tools, Taipei, Taiwan, KRT-BA06) were used for cDNA synthesis. The expression of target genes was detected via real-time PCR performed on a StepOnePlusTM system (Applied Biosystems, Waltham, MA, USA) with SYBR Green Master Mix (Applied Biosystems, Waltham, MA, USA, 4385612). The genes included ULK1, ATG2A, ATG2B, ATG3, ATG4B, ATG5, BECN1, ATG7, GABARAP, GABARAPL1, GABARAPL2, MAP1LC3B, ATG9, ATG10, ATG12, ATG13, ATG14, ATG16L1, ATG16L2, RB1CC1, WIPI1, SNX30, SNX4, ATG101, SQSTM1 and GAPDH. The primer sequence for each gene is shown in Table 1.

### 2.3. Cell Viability Assay

The cells were seeded at a concentration of 5x10^3^ cells/well in 96-well plates (37 °C and containing 5% CO2) and silenced with siRNA against GABARAPL1 (Ambion, Life Technology, Carlsbad, CA, USA). Cell viability was determined via the CellTiter-Glo^®^ Luminescent Assay kit (G7572, Promega, Madison, USA) after the ARPE-19 cells were treated with H_2_O_2_ for 24, 48, 72, and 96 h. Cell viability was determined based on the luminescent signal of cellular ATP.

### 2.4. Transfection 

For GABARAPL1 expression vector transfection, HA-GABARAPL1 vector kindly provided by Carol Mercer (addgene, 137759) was mixed with Lipofectamine 2000 (Invitrogen, 11668-027) for 30 min and then transfected into cells overnight for further experiments. For small interfering RNA (siRNA) transfection, ARPE-19 cells were transfected with RNAiMAX (Life Technologies, 13778-150) and 5 nM of off-targeting siRNA (Life Technologies, Carlsbad, CA, USA; 12935-112) or siRNA against GABARAPL1 (Dharmacon, Lafayette, USA). The cells were then exposed to H_2_O_2_ for 6 h or 96 h and harvested for protein expression analysis through immunoblotting.

### 2.5. Autophagic Flux Measurement and Immunoblotting

The ARPE-19 cells were treated with siRNA against GABARPL1 and exposed to H_2_O_2_ for 6 h or 96 h in the presence or absence of the autophagy inhibitor chloroquine (CQ) (20 μM; Sigma–Aldrich, St. Louis, USA; C6628) [17]. Briefly, the cells were harvested and lysed with 200 μL of radioimmunoprecipitation assay buffer (RIPA) buffer in the presence of 1% NP40, 150 mM of NaCl, 0.1% sodium dodecyl sulfate (SDS; Calbiochem, New Jersey, USA, 428015), 0.25% sodium deoxycholate (Sigma–Aldrich, St. Louis, USA; D6750), 50 mM of Tris-HCl, pH 7.5, and a protease inhibitor cocktail (Roche, Basel, CH, 11873580001). The proteins of lysed cells were separated through SDS-PAGE and transferred onto Nitrocellulose (NC) membranes. Next, the membranes were incubated with primary antibodies against GABARAPL1 (Protein Technology, 11010-1-AP), SQSTM1 (BD Pharmingen, 610832), ATG4B (Cell Signaling, 5299S), HA-tag (Cell Signaling, 3724), MAP1LC3B (Sigma–Aldrich, L7543) and actin (Cell Signaling, 4970S) at 4 °C overnight. The proteins were used to quantify the accumulation of MAP1LC3B-II to assess autophagic flux. Briefly, MAP1LC3B-II protein in cells was normalized with internal control actin. The net change of MAP1LC3B between DMSO and CQ treatment was used as the basal level of the autophagic flux (fold:1). The MAP1LC3B protein level in combinational treatment of H_2_O_2_ and CQ deducted MAP1LC3B protein in cells and then normalized with the basal level of the autophagic flux to determine if H_2_O_2_ modulates autophagy activity. Target proteins were incubated with horseradish peroxidase (HRP)-labeled secondary antibody (Santa Cruz, sc-2004 or sc-2005) and measured using electrochemiluminescence (ECL) reagent. The protein level was analyzed with the ChemiDoc XRS Imaging System (Bio-Rad, Hercules, CA, USA).

### 2.6. Statistical Analysis

All data are expressed as the mean ± standard error of the mean (SEM) of at least three independent experiments. The Mann–Whitney U-test nonparametric 2-tailed Student’s *t*-test was used to perform statistical analysis with Prism 5.0 (GraphPad, San Diego, CA, USA) to compare the effects between each group. *p* values < 0.05 were considered significant (* *p* < 0.05, ** *p* < 0.01, *** *p* < 0.001).

## 3. Results

**Figure 1:**
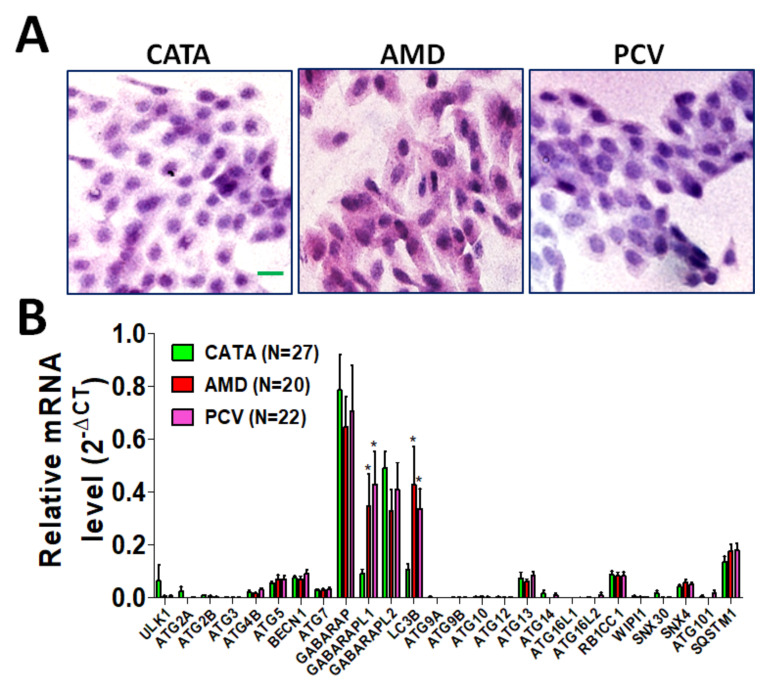
Comparison of autophagy-related genes in different diseases. Seventy samples were obtained from patients with cataracts (CATA, *n* = 27), neovascular age-related macular degeneration (AMD, *n* = 20) and polypoidal choroidal vasculopathy (PCV, *n* = 22) and assessed by using conjunctiva impression cytology. (**A**) The cells were stained by hematoxylin and eosin to observe the cellular and nuclear morphology. Scale bar: 20 µm. (**B**) The mRNA levels of autophagy-related genes were determined by real-time PCR. The results were analyzed using Prism 5.0. The quantitative results are expressed as the mean ± SD. * *p* < 0.05 AMD vs. CATA or PCV vs. CATA.

To examine if autophagy-related (ATG) gene expression is associated with patients with AMD and PCV, the cells obtained by impression cytology were stained to observe cellular morphology (Figure 1A). The cell morphology from patients with CATA and PCV was round, whereas the cell morphology was a little sharper in patients with AMD. Further, the RNA extracted by impression cytology showed that both the nAMD and PCV groups had significantly higher GABARAPL1 and MAP1LC3B mRNA levels than the controls, but there was no significant difference between the AMD and PCV groups (nAMD vs. control *p* = 0.02; PCV vs. control *p* = 0.01; nAMD vs. PCV *p* = 0.70) (Figure 1B). Moreover, GABARAPL1 gene expression in patients with AMD was significantly higher than that in patients with cataracts as our control group (*p* = 0.013, Table 2). GABARAPL1 gene expression in younger AMD patients was significantly higher compared to the AMD patients with age > 60 (≤60 vs. >60, *p* = 0.007, Table 2). Nevertheless, there was no significant association between GABARAPL1 expression and sex.

**Figure 2:**
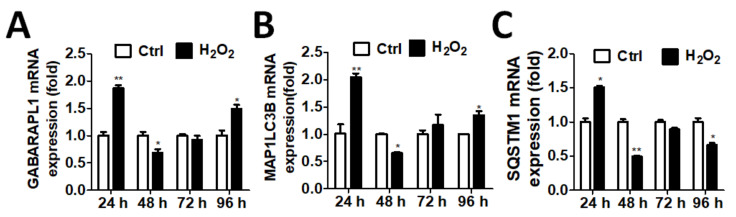
The mRNA levels of autophagy-related genes in adult retinal pigment epithelial (ARPE-19) cells during oxidative stress. ARPE-19 cells were treated with H_2_O_2_ (250 µM) for 24 h. The mRNA levels of (**A**) gamma-aminobutyric acid receptor-associated protein-like 1 (GABARAPL1), (**B**) microtubule-associated proteins 1A/1B light chain 3B (MAP1LC3B) and (**C**) SQSTM1 were determined by real-time PCR. The results were analyzed using Prism 5.0. The quantitative results are expressed as the mean ± SD from three independent experiments. * *p* < 0.05, ** *p* < 0.01 Cells treated with H2O2 vs. cells without treatment.

To further examine whether oxidative stress causes ATG expression, as observed in patients, we tested the effects of the above two genes in ARPE-19 cell lines. The results showed that the GABARAPL1 and LC3 mRNA expression was increased significantly when the ARPE-19 cells were treated with 250 µM of H_2_O_2_ for 24 h (Figure 2A,B). The SQSTM1 mRNA expression showed a similar trend (Figure 2C).

**Figure 3:**
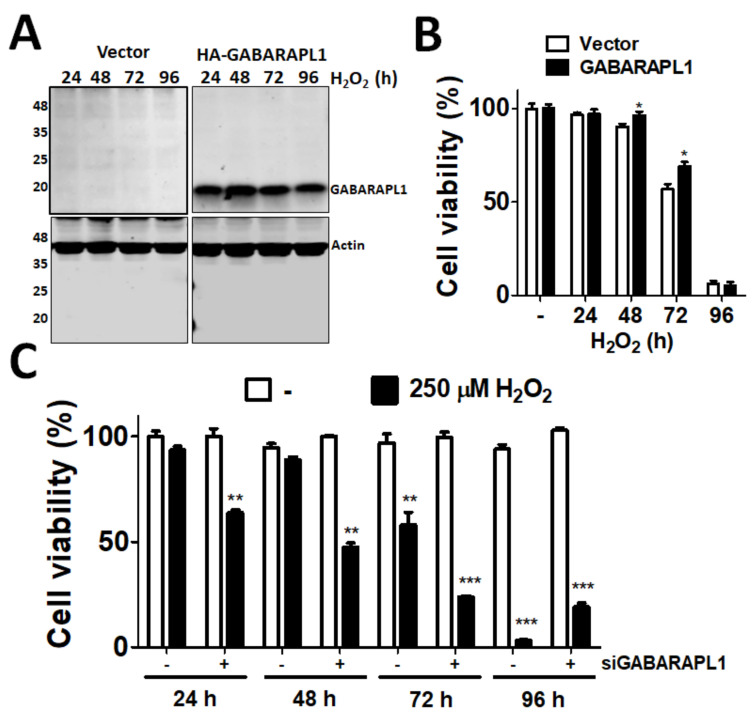
The effects of GABARAPL1 on ARPE-19 cell viability during oxidative stress. (**A**) Human ARPE-19 cells were transfected blank vector or vector encoding HA-GABARAPL1 and treated with H_2_O_2_ (250 μM) for 24, 48, 72, and 96 h. The protein levels of GABARAPL1 and Actin were determined by immunoblotting. Molecular weight is shown in the left-hand side of the blot. (**B**) The cells viability was examined by a Cell-titer Glo^®^ Assay System. (**C**) ARPE-19 cells were transfected 5 nM of off-targeting siRNA (−) or siRNA against GABARAPL1 ( + ) and exposed to H_2_O_2_ (250 μM) for 24, 48, 72, and 96 h. Cell viability of the cells was measured with a Cell-titer Glo^®^ Assay System. The data were analyzed with Prism 5, and the results are shown as the mean ± SEM from three independent experiments. ** *p* < 0.01, *** *p* < 0.001 siGABARAPL1 vs. non-targeting control siRNA (siCtrl).

To determine the effect of GABARAPL1 on oxidative stress in ARPE-19 cells, the cells were transfected with empty vector or vector encoding GABARAPL1 and treated with H_2_O_2_ for different time points (Figure 3A,B). The cells ectopically expressing GABARAPL1 were more viable at 48 h and 72 h after exposure to oxidative stress compared to the cells with blank vector. Furthermore, we silenced GABARAPL1, and the cells were then exposed to H_2_O_2_ for different durations. The results showed that the cell viability of the GABARAPL1-silenced cells was significantly lower than that of the control cells at 24, 48, and 72 h. However, the cell survival rate after 96 h of exposure to H_2_O_2_ in the non-silenced control group decreased to as low as 5%, but the cell viability was maintained at a certain level in the knockdown group (Figure 3). Our results suggested that GABARAPL1 probably played a different role in short-term and long-term oxidative stress.

**Figure 4:**
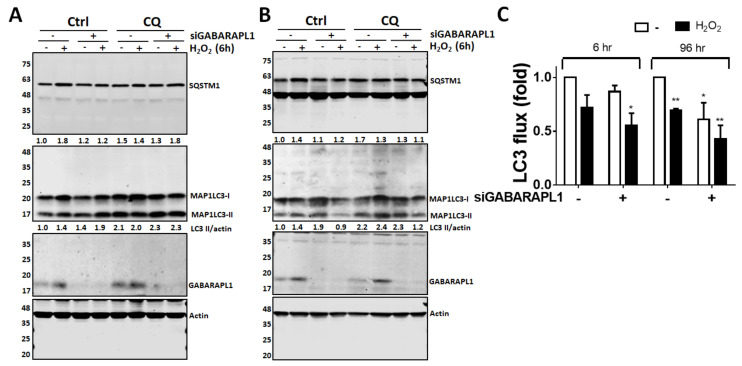
The effect of GABARAPL1 on autophagic flux in ARPE-19 cells exposed to oxidative stress. ARPE-19 cells were transfected with 5 nM of siRNA against GABARAPL1. The cells were then exposed to 250 µM of H2O2 for 6 h (**A**) or 96 h (**B**). The cells were lysed for Western blots using an antibody against MAP1LC3B to analyze the autophagic flux level. Molecular weight is shown in the left-hand side of the blot. (**C**) The quantitative results of autophagic flux in ARPE19 during oxidative stress are shown as the mean ± SD from three independent experiments. * *p* < 0.05, ** *p* < 0.01 vs. cells with non-targeting control siRNA (−) and without H_2_O_2_ (−).

To further investigate the effects of GABARAPL1 in the ARPE19 cells exposed to oxidative damage, we treated ARPE-19 cells with siRNA against GABARAPL1 and then exposed them to H_2_O_2_ for 6 or 96 h. The autophagic flux decreased after exposure to H_2_O_2_, especially in those with siRNA against GABARAPL1. Silencing GABARAPL1 itself caused a reduction in the autophagic flux compared with that of the control only group at 96 h, but the damage caused by oxidants was comparatively ameliorated. The results suggested that autophagy might be protective against short-term oxidative stress but harmful against long-term oxidative stress (Figure 4).

## 4. Discussion

Our study is the first to investigate autophagy-related gene expression in patients with age-related macular degeneration by using conjunctival impression cytology. Both the nAMD and PCV groups had significantly higher GABARAPL1 and MAP1LC3B mRNA levels than the controls, but there was no significant difference between the AMD and PCV groups. There was no difference in sex. Age difference only existed in the AMD group. Therefore, even though the GABARAPL1 expression was significantly higher in AMD patients compared to that in CATA patients, the variability of gene expression from patients was high, likely due to individual difference and severity in each patient. However, the sample size was not enough to see the correlation of gene expression with clinicopathological outcome, which need more study to elucidate.

Further in vitro studies revealed that GABARAPL1 and MAP1LC3B mRNA expression increased significantly after acute oxidative stress in ARPE-19 cells. However, the cell viability change in the GABARAPL1-silenced cells was different after short- and long-term oxidative stress. Elevated GABARAPL1 was beneficial in ARPE-19 cells during short-term oxidative stress. After long-term oxidative stress, the cell viability was increased in the GABARAPL1-silenced cells. Our results suggested that GABARAPL1 probably played different roles in short-term and long-term oxidative stress. Silencing GABARAPL1 itself caused a reduction in autophagic flux compared with that of the control group at 96 h, but the oxidative damage caused by oxidants was comparatively ameliorated. The results suggested that autophagy might be protective in short-term oxidative stress but harmful in long-term oxidant exposure.

At present, autophagy is generally regarded as a regulatory mechanism of defense and a survival response to stress. However, autophagy plays a complicated role in cells. This process can improve cell survival through the recycling of proteins but inhibiting autophagy can induce cell death through the activation of apoptosis in RPE cells. The majority of reports support the protective role of autophagy [18,19,20]. However, increasing evidence implicates autophagy in stress-induced cell death in certain settings [13,21,22,23]. GABARAPL1, one of the most important autophagic genes that can promote the fusion of autophagosomes and lysosomes to form autolysosomes, was significantly higher in eyes with AMD than in control eyes in our gene analysis by conjunctiva impression cytology. Moreover, our in vitro study suggested a possible discrepancy in the self-defense effect between long-term and short-term oxidative injury. Persistent chronic inflammation has been proposed to be the cause of age-related macular degeneration or diabetic retinopathy [24,25]. Dysregulated autophagy might result in pathological changes. The discrepancy between different studies might be due to the duration of damage in the model or the stage of disease. The underlying mechanism or critical factors require further investigation to confirm this hypothesis.

Impression cytology is an established technique for ocular surface cell evaluation and protein biomarker quantification [16]. Most studies focused on ocular surface disorders; our study is the first to assess the autophagy-related gene expression of patients with age-related macular degeneration. Most studies use aqueous or vitreous samples to represent the status of retina, however, it is an invasive approach. Recent study suggests the possibility of tear film as a non-invasive predictor test for the severity of diabetes mellitus (DM) [26]. Compared to tear film, conjunctiva impression cytology may offer more cell sampling and is also non-invasive. Therefore, we proposed this study. We showed that both the GABARAPL1 and MAP1LC3B mRNA levels were significantly higher in AMD cases than in controls. Our results suggested the speculation that autophagy might play a role in the pathogenesis of AMD. Nevertheless, there is no direct relation between the conjunctival epithelia and RPE cells. It is ethically and technically challenging to obtain AMD RPE cell specimens. The precise role of GABARAPL1 in AMD pathogenesis and the potential use of impression cytology in AMD require further investigation. 

In summary, our study showed the possibility of assessing autophagy-related gene expression in patients with age-related macular degeneration by using conjunctival impression cytology. GABARAPL1 was significantly higher in eyes with AMD than in control eyes. Although an in vitro study showed an initial protective effect of autophagy, a cell viability study revealed the possibility of a harmful effect after long-term oxidative injury. However, our present study showed the effects of GABARAPL1 in ARPE-19 cells, which only partially resembles the biology, transcriptome, and proteome of primary RPE cells. Therefore, the underlying mechanism or critical factors require further investigation to prove this hypothesis. 

## Figures and Tables

**Table 1:** Primer sequences for the real-time polymerase chain reaction quantification of human autophagy-related gene transcripts.

Gene Symbol	Forward Primer (5′→3′)	Reverse Primer (5′→3′)
ULK1	TGCCCCTGGTTGAATGTTCT	ACACCAGCCCAACAATTCCA
ATG 2A	GCCTGGCGCCTAGAGAAG	ACCCGCTCTTTCACACAGTT
ATG 2B	CGTTGTCAGTTCAGCCAAGA	AGATGTGGCAAACCACGGAT
ATG 3	TCCCATGTGTTCAGTTCACCC	AACAGCCATTTTGCCACTAATC
ATG 4A	AGTCAGAGTAAGGGCACCTCT	CCCCTAAAGACTGTGGCATCT
ATG 4B	TCCATCGCTGTGGGGTTTTT	AGAATCTAGGGACAGGTTCAGGA
ATG 4C	TTACTACGGTGGCCGGGGT	AAAAATGTGCAGGAGCCACCAA
ATG 4D	AACGTCAAGTACGGTTGGGT	ACACAAAGTCCCGCTGGAAA
ATG 5	AGACCTTCTGCACTGTCCATC	GCAATCCCATCCAGAGTTGCT
BECN1	AACCAGATGCGTTATGCCCA	TCCATTCCACGGGAACACTG
ATG 7	AGCGGCGGCAAGAAATAATG	GTCCTTGGGAGCTTCATCCA
GABARAP	GAGGGCGAGAAGATCCGAAA	AGCTCGGAGATGAATTCGCTT
GABARAPL1	TGAGACCTGAGGACGCCTT	GGGCTTCCAACCACTCATTTC
GABARAPL2	GCGAAATATCCCGACAGGGT	CCACAAACAGGAAGATCGCC
MAP1LC3B	AAGGCTTTCAGAGAGACCCTG	CCGTTTACCCTGCGTTTGTG
ATG 9A	GCTGTTCCTGAGGTGGTCAA	TATGGTGCCAAGGTGACTTGC
ATG 9B	CTCTAGCCCCAGACAACAGTG	TACTCCACCCTCCAATCTCCT
ATG 10	CGAGCGGAGAGGGTTATCATT	GCACATGTAGCCATCAGAACAG
ATG 12	CGGATGTCTCCCCAGAAACAA	CCTTGGATGGTTCGTGTTCG
ATG 13	CCGAAAAGTGGGGGCTTTTG	TCGGTATCCTCCAGCTCCAA
ATG 14	GCTCTCCTCTCAGGCCATCAT	TCTGAACGCATTTGGCGCA
ATG 16 L1	TCGAGGAGATCATCCTGCAATAAC	TCAGTTGGGCCATTTCTTGTAGC
ATG 16 L2	GCCAAACAGTGTCACTCCCA	AAGCAAGCTCACCACAGACC
RB1CC1	CATCCTAGACGAACGCCATGA	TGGTTTGAGATCCAGGGCAG
WIPI1	TCCACGGAAGCAATGAAATCC	TAGGCAAACCAGCAGCCTTT
SNX 30	TGGAGAGGTGGCAGAACAAC	GAATAATCGACTCCCACGCCA
SNX 4	GTTTTCAGTGAATGGAGTGCC	TTCATGTTTCCTGCACACAGC
ATG 101	TGTTTTAACCGTGTGCCCCCT	ACAAGAGACCAGCTCCACAGT
SQSTM1	CTGCCCAGACTACGACTTGTGT	TCAACTTCAATGCCCAGAGG
GAPDH	TGCACCACCA ACTGCTTAGC	GGCATGGACT GTGGTCATGA G

**Table 2:** Comparison of GABARAPL1 expression between age-related macular degeneration and normal control.

Variable	%	Control (*n* = 27)			%	AMD (*n* = 42)			*p* Value *
		Mean ± SD	Median	*p* Value *		Mean ± SD	Median	*p* Value *	
**Total**		0.0850 ± 0.0495	0.0773			0.3057 ± 0.4457	0.0972		0.013
**Sex**									
**Female**	66.67%	0.0923 ± 0.0478	0.0808	0.284	45.20%	0.2845 ± 0.3971	0.0813	0.781	
**Male**	33.33%	0.0702 ± 0.0525	0.0829		54.80%	0.3241±0.4924	0.0983		
**Age, y**									
**≦60**	25.93%	0.0794 ± 0.0503	0.0864	0.739	14.30%	0.7504 ± 0.5594	0.6178	0.007	
**>60**	74.07%	0.0868 ± 0.0504	0.0773		85.70%	0.2295 ± 0.3832	0.0886		

Abbreviations: SD, standard deviation. * *p* values were estimated by Student’s *t*-test.

## Abbreviations

PCV	polypoidal choroidal vasculopathy
ERBB2	erb-b2 receptor tyrosine-protein kinase 2
GABARAPL1	Gamma-aminobutyric acid receptor-associated protein-like 1
AMD	age-related macular degeneration
ATG	autophagy-related gene
SQSTM1/p62	Sequestosome 1
MAP1LC3B	Microtubule-associated proteins 1A/1B light chain 3B
